# Dose delivery reproducibility for PBS proton treatment of breast cancer patients with and without mask immobilization

**DOI:** 10.1186/s13014-023-02323-3

**Published:** 2023-09-22

**Authors:** Yixiu Kang, Martin Bues, Michele Y. Halyard, Lisa A. McGee, Tamara Z. Vern-Gross, William W. Wong, Sameer R. Keole, Carlos Vargas, Sarah E. James, Safia K. Ahmed, James P. Archuleta, Ana K. Ridgway, Pedro R. Lara, Mirek Fatyga

**Affiliations:** Department of Radiation Oncology, Mayo Clinic, 5881 East Mayo Blvd, Phoenix, AZ 85054 USA

**Keywords:** Proton therapy, Pencil beam scanning proton therapy, Breast proton therapy immobilization, Breast and post-mastectomy chest wall proton radiation therapy

## Abstract

**Background:**

Setup reproducibility of the tissue in the proton beam path is critical in maintaining the planned clinical target volume (CTV) dose coverage and sparing the organs at risk (OAR). In this study, we retrospectively evaluated radiation therapy dose reproducibility for proton pencil beam scanning (PBS) treatment of breast cancer patients with and without mask immobilization.

**Methods:**

Ninety-four patients treated between January 2019 and September 2022 with at least one verification CT scan (V-CT) in treatment position were included for this study. All patients were set up with arms up using the Orfit AIO patient positioning system, with (69 patients) or without (25 patients) mask immobilization in chin, neck, shoulder, upper arm, and chest areas. Two to three enface or near enface single field uniform dose PBS beams were optimized using a commercial treatment planning system. Prescription doses were 25 to 60 Gy_RBE_ in 5 to 45 fractions. Treatment plan doses re-calculated on V-CTs were compared to the corresponding planned doses. Cumulative doses were also calculated for patients with at least 3 V-CTs by deform and weighted sum doses from V-CTs to corresponding P-CTs. CTV D95%, ipsilateral-lung V40%, esophagus D0.01cc, and heart mean dose were evaluated and reported as percentages of prescription doses. Differences were large dose deteriorations (LDD) if: (1) CTV (V-CT/cumulative D95%) – (Planned D95%) < − 5%; or (2) Ipsilateral-lung (V-CT/cumulative V40%) – (Planned V40%) > 5%; or (3) Esophagus (V-CT/cumulative D0.01cc) – (Planned D0.01cc) > 10%; or (4) Heart (V-CT/cumulative mean) – (Planned mean) > 1.5%.

**Results:**

On average, V-CT/cumulative and planned CTV/OAR dose parameter differences were less than 2.2%/1.7% and 3.4%/3.7% for masked and maskless patients, respectively. The percentages of patients with at least one CTV or OAR V-CT/cumulative dose LDD were 20.3%/25.0% and 72.0%/54.0% for masked and maskless patients, respectively.

**Conclusions:**

On average, masked/maskless setups achieved delivered and planned CTV/OAR dose parameters agreed within 2.2%/3.7% for PBS treatment of breast cancer patients in this study. Maskless patients had higher rate of CTV/OAR LDDs compared to masked patients. Dosimetric differences large enough to raise clinical concerns in either group were able to be addressed with replannings.

## Background

Radiation therapy reduces the risk of loco-regional recurrence and breast cancer mortality for breast cancer patients after breast conserving surgery or mastectomy [1, 2]. Proton therapy can achieve improved target coverage and critical organ sparing compared to photon treatment for breast cancer patients in planning studies [3–6], and has been used in treating breast cancer patients with acceptable toxicity [7–11].

Treating breast cancer patients with proton pencil beam scanning (PBS) is challenge because proton beam range is sensitive to the setup reproducibility of the tissue in the beam paths. Soft tissue in the breast and neck areas could deform and move, which could result in a tissue difference in the beam paths. The tissue difference in beam path could cause large dose difference to the distal portion of the target and/or to the organs at risks (OAR) distal to the target such as lung, heart, and esophagus. To date, as far as we know, there are no inter-fractional dose reproducibility studies comparing PBS treatment of breast cancer patients with and without mask immobilization. In this work, we retrospectively evaluated the dose delivery reproducibility, i.e., the closeness of the agreement between the delivered and planned dose, for PBS breast cancer patients treated with and without mask immobilization. The delivered dose in this study is the dose distribution calculated on verification simulation computed tomography (V-CT) scan in treatment position.

## Methods

### Patient selection

This study was exempted by our institution research board (IRB) 22-001077. Ninety-four breast cancer patients treated in our department between January 2019 and September 2022 with proton therapy that had at least one V-CT during proton therapy were included for this study. All post-mastectomy chest wall (CW) patients treated before February 2021 and all breast patients treated before August 2021 were immobilized with thermoplastic mask (Orfit Industries America, Norfolk, Virginia), and the remainder of the patients had no mask immobilization. The change of immobilization method was to converge proton radiation therapy setup to photon radiation therapy setup. Currently, we only use mask immobilization for selected bilateral and large breast patients treated with proton. There were 69 masked patients receiving 96 V-CTs and 25 maskless patients receiving 65 V-CTs. Among these patients, 8 masked patients and 13 maskless patients had at least 3 weekly V-CTs during the proton therapy treatment course. Patients and target information are listed in Table 1. Prescription doses ranged from 25 to 60 Relative-Biological-Effectiveness dose in Gray (Gy_RBE_) in 5 to 45 fractions.

**Table 1:** Patient and target information

	With mask	No mask
*Age*		
Median (range)	67 (36, 85)	65 (32, 83)
Number of patients	69	25
Breast	43	9
CW	26	16
*Laterality*		
Left	38	20
Right	25	4
Bilateral	6	1
*Nodal regions treated*		
Axilla	57	20
SCV	52	20
IMN	51	19
*Breast/CW Rx (cGy*_*RBE*_*)*		
5 × (500 or 520)	4	8
15 × 267	36	14
25 × (180 or 200)	21	2
Other	8	1
SIB	34	6
*CTV Volume (cc)*		
Median (range)		
Boost	38.3 (4.2, 463.0)	92.6 (5.3, 749.9)
Breast/CW	685.1 (163.6, 2599.5)	671.1 (142.3, 1808.7)
Axilla	116.5 (40.6, 343.4)	136.7 (45.0, 289.1)
SCV	23.4 (10.3, 69.8)	27.6 (10.3, 54.7)
IMN	6.0 (1.0, 27.1)	6.0 (1.5, 15.3)

CW, chest wall; SCV, supraclavicular nodes; IMN, internal mammary nodes; Rx, prescription; Gy_RBE_, Relative-Biological-Effectiveness dose in Gray; SIB, simultaneous integrated boost; CTV, clinical target volume

### Patient immobilization and CT simulation

**Fig. 1:**
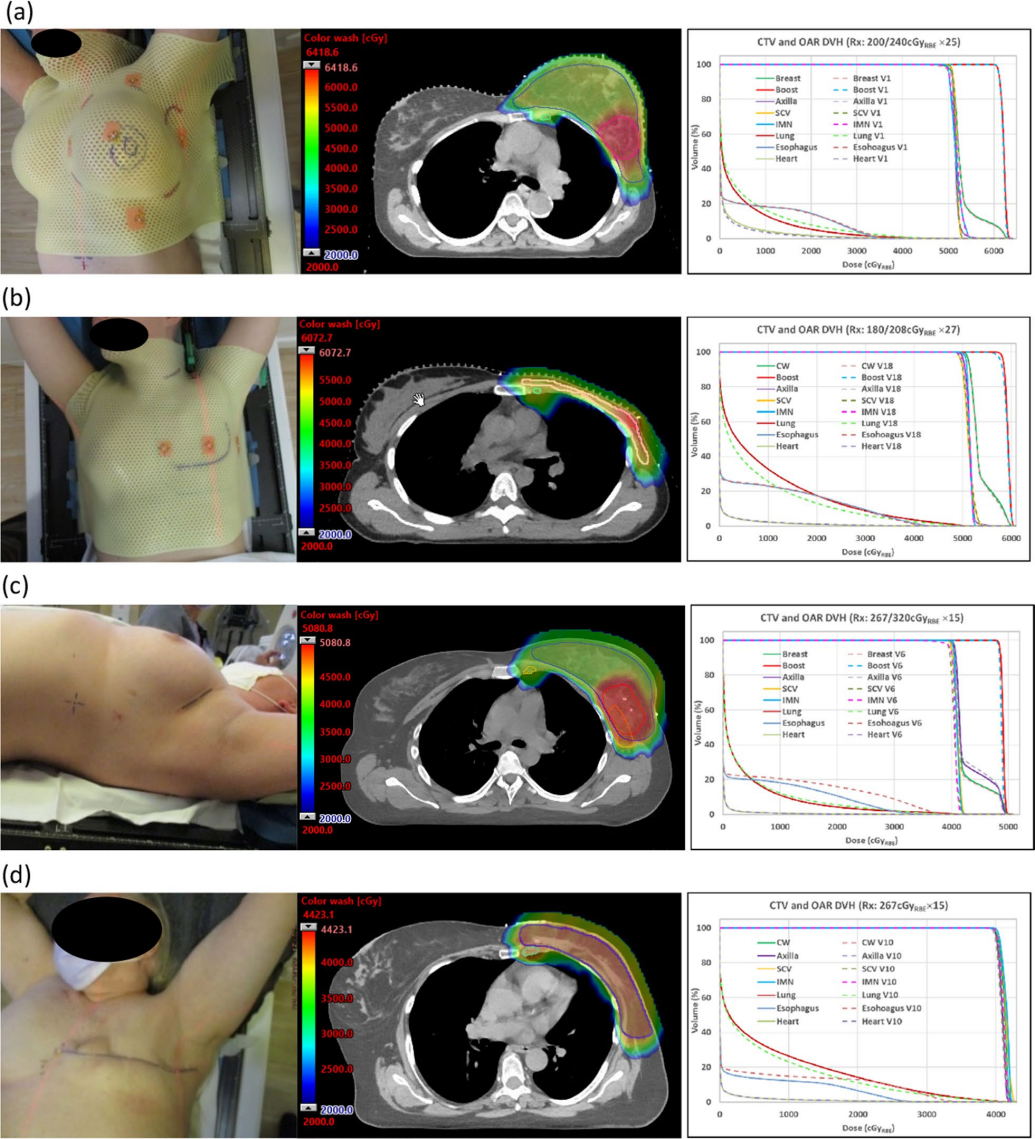
Patient setup, treatment plan dose and DVH for **a** a masked breast patient, **b** a masked CW patient, **c** a maskless breast patient, and **d** a maskless CW patient. Abbreviations: CTV, clinical target volume; OAR, organ at risk; DVH, dose volume histogram; Rx, prescription; Gy_RBE_, Relative-Biological-Effectiveness dose in Gray; CW, chest wall; Vx, verification Computed Tomography at fraction x; SCV, supraclavicular node; IMN, internal mammary nodes

All patients were setup in supine position with both arms up using Orfit AIO breast and lung board (Orfit Industries America, Norfolk, Virginia) (Fig. 1). Cushion set and head rest were used. Patients’ heads were turned away from the treatment side with the chins extended. Heads were midline for patients with bilateral disease. For masked patients, 4-point thermoplastic masks were fabricated before CT simulation to immobilize patient. The two lower mask fixation points were secured at the two slots on the patient side in thoracic area. The upper mask fixation point opposite to the treatment side was secured to the slot between the patient neck and upper arm. The upper mask fixation point on the treatment side was secured to the slot outside of the patient upper arm with an extra fixation point added in between the patient’s neck and upper arm to immobilize the shoulder and upper arm. When the mask was fabricated, the soft tissue in the patient neck, shoulder and breast areas on the treatment side were manipulated as needed to reduce skin folds and to make the breast a smooth protrusion shape. Patients were instructed to do abdominal or shallow breathing near end of expiration when the mask was fabricated. For patients with intact breast and patients with implants, four crescent lines were marked on patient’s skin around the breast and covered with Tegaderm. Once mask was hardened, nipple, scar and crescent lines were drawn on the mask corresponding to the location on patient skin. Two lines 2.5-mm on each side of the scar and crescent lines were also drawn on the mask. Five BBs were placed on the mask for patients with intact breasts and patients with implants, and three BBs were placed on the mask for patients receiving post-mastectomy CW irradiation. Before performing CT scan for the patient, the mask was removed and reapplied to check reproducibility for treatment setup so that: 1. no airgap between patient and mask anywhere in the chin, neck, shoulder, and chest areas on the treatment side; and 2. nipple was aligned with the mark on mask; 3. scar and crescent lines on patient were aligned with those marked on the mask within 2.5-mm. All patients were scanned using a Siemens Somatom Definition AS Open RT CT scanner (Siemens Medical, Erlangen, Germany) with 2-mm slice thickness.

### Treatment plan, treatment setup, plan delivery and V-CT

Each treatment plan was optimized using 2 to 3 enface or near enface single field uniform dose proton PBS beams on planning simulation CT (P-CT) in Eclipse treatment planning system (Varian Medical System, Palo Alto, CA). The proton beam gantry angles ranged from 0 to 60 degrees with at least 25-degree separation for beams in a same plan. Plan optimization goals were as follows: 1. Clinical target volume (CTV) dose to 95% volume (D95%) ≥ 95% of the prescription dose using 5-mm setup and 3% range uncertainty dose evaluation; 2. Ipsilateral-lung volume receiving 40% of the prescription dose (V40%) ≤ 15–20% of the prescription dose; 3. Dose to 0.01cc (D0.01cc) esophagus volume ≤ 72–90% of the prescription dose; 4. Mean heart dose ≤ 1.5–3.0% of the prescription dose; 5. Dose to 5-mm/3-mm skin for breast/CW ≤ 100% of prescription dose; and 6. Overall max dose ≤ 107% of prescription dose. Compromise would be made by physician discretion if above goals could not be achieved.

The proton treatment was delivered using the Hitachi Probeat-V (Hitachi, Ltd., Tokyo, Japan) PBS proton beam delivery system [12]. All patients were aligned using two orthogonal planar kV x-ray images for each treatment so that: (1) The surgical clips, ribs, and spine were aligned within 5-mm to P-CT digitally reconstructed radiographs (DRRs); (2) The supraclavicular bone and humeral head on the treatment side were aligned within 3-mm to P-CT DRRs; and (3) The BBs on x-ray images were aligned within 3-mm to P-CT DRRs for masked patients. For masked patients, the mask was applied on patient and marks on skin and mask were aligned within 2.5-mm before x-ray image setup.

The V-CT scans were acquired at treatment position using the same type of CT scanner as used for the planning CT scans. All V-CTs were acquired using CT-On-Rails in the treatment room after x-ray setup except 16.6% of the V-CT for masked patients were acquired in CT sim room. Each V-CT was registered to corresponding P-CT using bony and/or BB alignment simulating treatment setup to calculate dose distribution on V-CT. CTVs were propagated from P-CT to V-CT using RayStation 9A to 11ASP2 (RaySearch Laboratories, Stockholm, Sweden) hybrid intensity and structure based deformable image registration. OARs were contoured by dosimetrists. CTVs and OARs were modified by physicians as needed. For patients with at least 3 V-CTs, cumulative doses were also calculated in RayStation using the same deformable image registration for CTV propagation to deform the dose distributions on V-CT to corresponding P-CT. The weighted sum of the deformed dose distributions on P-CT was used as cumulative dose to evaluate total delivered dose to patients. The weight of each V-CT dose was determined by assuming each V-CT dose represented the dose delivered to the patients for the fractions closest to the day the V-CT was taken and the sum of the weights of all V-CT doses was the total number of the treatment fractions. For patients with replans, the replanned dose distributions were also deformed to initial P-CT and weighted summed on initial P-CT to evaluate the total planned doses.

### Dose evaluation

The CTV D95%, ipsilateral-lung V40%, esophagus D0.01cc, and heart mean dose on V-CT doses were compared to those on the planning CTs to evaluate the delivered dose reproducibility. All dose parameters were evaluated as percentages of the prescription doses. The dose parameter differences between V-CT/cumulative and planned doses were calculated as the dose parameter evaluated on V-CT/cumulative dose distribution minus the same dose parameter evaluated on the corresponding planned dose distribution. Dose differences were considered to be large dose deteriorations (LDDs) if: 1. CTV (V-CT/cumulative D95%) – (Planned D95%) < − 5%; or 2. Ipsilateral-lung (V-CT/cumulative V40%) – (Planned V40%) > 5%; or 3. Esophagus (V-CT/cumulative D0.01cc) – (Planned D0.01cc) > 10%; or 4. Heart (V-CT/cumulative mean) – (Planned mean) > 1.5%.

## Results

### Planned doses and dose differences between V-CT and P-CT doses

**Fig. 2:**
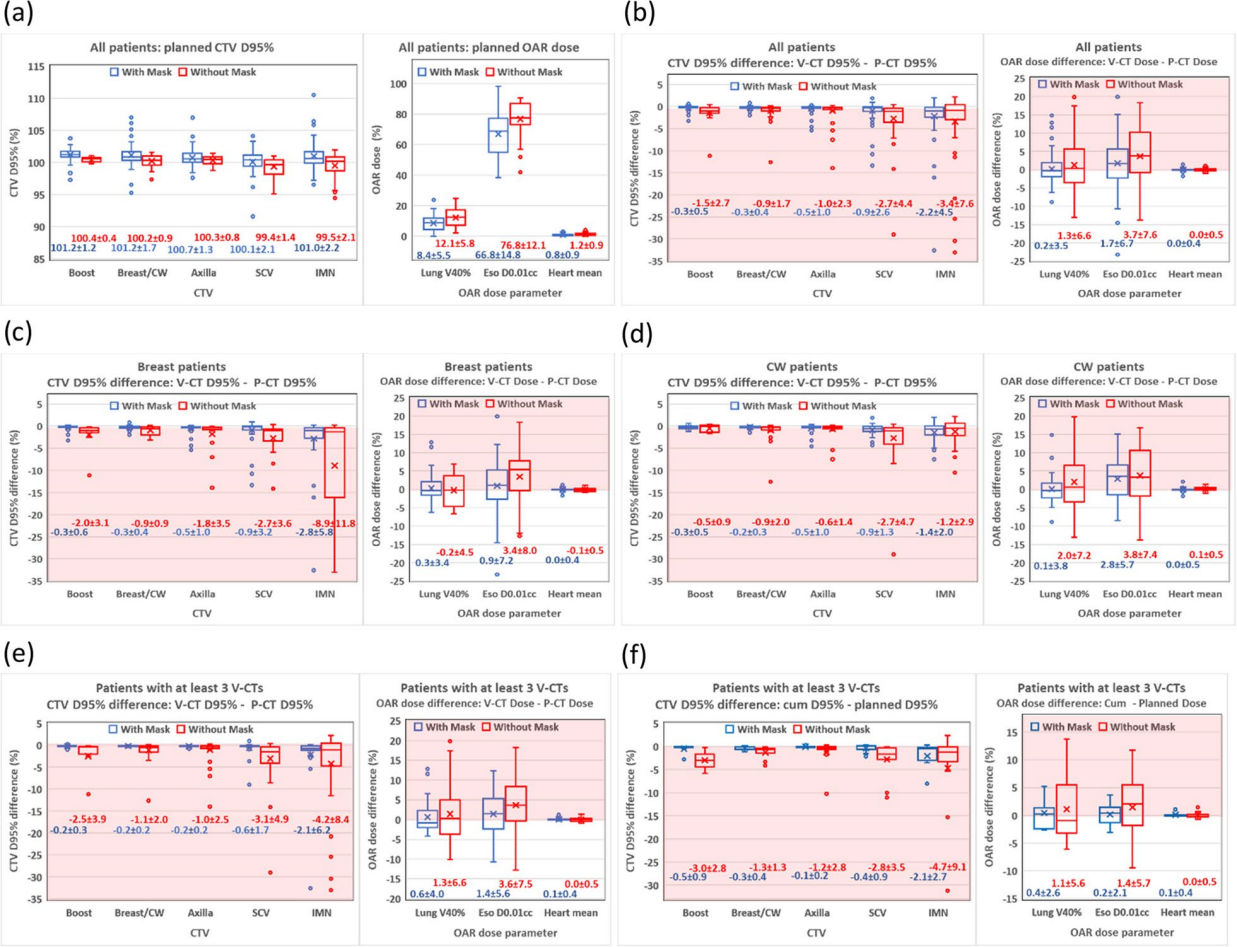
Box and whisker plots of CTV and OAR doses and dose differences for **a** and **b** all patients, **c** all breast patients, **d** all CW patients, and **e** and **f** all patients with at least three V-CTs. The horizontal lines and crosses in the boxes are the medians and averages. The circles are outlier points. The numbers blow the boxes are the mean ± 1SD. The pink shaded areas are the regions where CTV coverages or OAR doses were worse on V-CT/cumulative doses than those on planned doses. CTV, clinical target volume; CW, chest wall; SCV, supraclavicular node; IMN, internal mammary nodes; OAR, organ at risk; V-CT, verification computed tomography; P-CT, planning computed tomography; SD, standard deviation; cum, cumulative

Figure 2a shows the box and whisker plots of the P-CT CTV D95% and OAR doses for all patients. On average, masked patients had slightly higher planned CTV D95% and lower planned OAR doses than those of maskless patients.

Figure 2b shows the box and whisker plots of the V-CT and P-CT dose differences for all patients. The number of each CTV/OAR structure evaluated in the plot is listed in Table 2. On average, V-CT CTV D95% were lower than those of P-CT but were within 2.2% and 3.4% for masked and maskless patients, respectively. On average, V-CT ipsilateral-lung V40%, esophagus D0.01cc, and heart mean dose were higher than those of P-CT but were within 1.7% and 3.7% for masked and maskless patients, respectively. CTV and OAR dose parameter differences between V-CT and P-CT doses for masked patients showed smaller mean, median, 1 standard deviation (SD), and interquartile range than those for maskless patients.

**Table 2:** Percentage/number of LDDs for CTVs and OARs and the number of verification or cumulative doses evaluated for each structure

				CTV					OAR		
				Boost D95%	Breast/CW D95%	Axilla D95%	SCV D95%	IMN D95%	Ipsi-lung V40%	Esophagus D0.01cc	Heart mean
All patients	V-CT versus P-CT doses	With mask	LDD (%)	0.0	0.0	1.2	3.9	8.2	5.7	8.2	1.0
			V-CT (#)	63	105	82	77	73	105	73	96
		No mask	LDD (%)	6.7	1.5	7.3	16.7	20.8	31.8	27.3	0.0
			V-CT (#)	15	66	55	60	53	66	55	65
Breast patients	V-CT versus P-CT doses	With mask	LDD (%)	0.0	0.0	2.0	6.8	10.0	6.0	7.1	0.0
			V-CT (#)	42	68	50	44	40	67	42	61
		No mask	LDD (%)	10.0	0.0	11.8	17.6	40.0	23.8	23.5	0.0
			V-CT (#)	10	21	17	17	15	21	17	20
CW patients	V-CT versus P-CT doses	With mask	LDD (%)	0.0	0.0	0.0	0.0	6.1	5.3	9.7	2.8
			V-CT (#)	21	37	32	33	33	38	31	36
		No mask	LDD (%)	0.0	2.2	5.3	16.3	13.2	35.6	28.9	0.0
			V-CT (#)	5	45	38	43	38	45	38	45
Patients with at least 3 V-CTs	V-CT versus P-CT doses	With mask	LDD (%)	0.0	0.0	0.0	3.3	7.7	11.8	6.7	0.0
			V-CT (#)	31	34	30	30	26	34	30	30
		No mask	LDD (%)	16.7	2.3	7.5	20.0	22.5	25.6	22.5	0.0
			V-CT (#)	6	43	40	45	40	43	40	43
	Cum versus planned doses	With mask	LDD (#)	0	0	0	0	1	1	0	0
			V-CT (#)	8	9	8	8	7	9	8	8
		No mask	LDD (#)	1	0	1	2	3	4	1	0
			V-CT (#)	2	13	12	12	12	13	12	13

LDD, large dose deterioration; CTV, clinical target volume; OAR, organ at risk; CW, chest wall; SCV, supraclavicular nodes; IMN, internal mammary nodes; V-CT, verification computed tomography; P-CT, planning computed tomography; Cum, cumulative

Figure 2c, d show the box and whisker plots of V-CT and P-CT dose differences for breast patients and CW patients, respectively. The CTV and OAR dose difference for breast and CW patients agreed with those of all patients within statistical uncertainty.

### Dose differences between cumulative and planned doses

Figure 2e shows the box and whisker plots of V-CT and P-CT dose differences for patients with at least 3 V-CTs. The dose difference distributions of this sub-group of the patients are similar to those of all patients in this study (Fig. 2b). Therefore, this subgroup was a reasonable representation of the patients in this study.

Figure 2f shows the box and whisker plots of the cumulative and planned dose differences for patients with at least 3 V-CTs. On average, CTV D95% for cumulative doses were lower than planned doses but agreed within 2.1% and 4.7% for masked and maskless patients, respectively. On average, ipsilateral-lung V40%, esophagus D0.01cc, and heart mean dose for cumulative doses were higher than planned doses but were within 0.4% and 1.4% for masked and maskless patients, respectively.

### Patients with LDDs

Table 2 lists the percentage/number of CTVs/OARs showing LDDs on V-CT/cumulative doses compared to planned doses. Overall, patients had at least one CTV or OAR V-CT dose LDD were 20.3% (14 out of 69) and 72.0% (18 out of 25) for masked and maskless patients, respectively. For patients with at least three V-CTs, 25.0% (2 out of 8) and 54.0% (7 out of 13) had at least one CTV or OAR cumulative dose LDD for masked and maskless patients, respectively. Five of the 69 masked patients had replans and three of those had LDDs. Three of the 25 maskless patients had replans and all had LDDs. Two of the five masked patients with replans and three maskless patients with replans had at least 3 V-CTs.

In this study, no masked patients had boost or breast/CW CTV D95% V-CT dose LDDs. One maskless patient had boost CTV D95% LDD, and another maskless patient had breast/CW CTV D95% LDD. The percentage of V-CT doses with LDDs for axilla, supraclavicular node (SCV) and internal mammary node (IMN) D95% were 1.2%, 3.9%, and 8.2% for masked patients, and 7.3%, 16.7% and 20.8% for maskless patients, respectively. V-CTs with ipsilateral-lung and esophagus LDDs were 5.7% and 8.2% for masked patients, and were 31.8% and 27.3% for maskless patients, respectively. There was one V-CT dose showed heart LDD for masked patients while no maskless patients had heart LDD.

The dose differences between cumulative and planned dose showed one masked patient had LDD for IMN D95% and another masked patient had LDD for ipsilateral-lung V40%. Three maskless patients had at least one cumulative dose CTV D95% LDDs and another four maskless patients had cumulative ipsilateral-lung V40% and/or esophagus D0.01cc LDDs. The cumulative dose and planned dose in this study considered the replans.

### Causes of LDDs and methods to improve

**Fig. 3:**
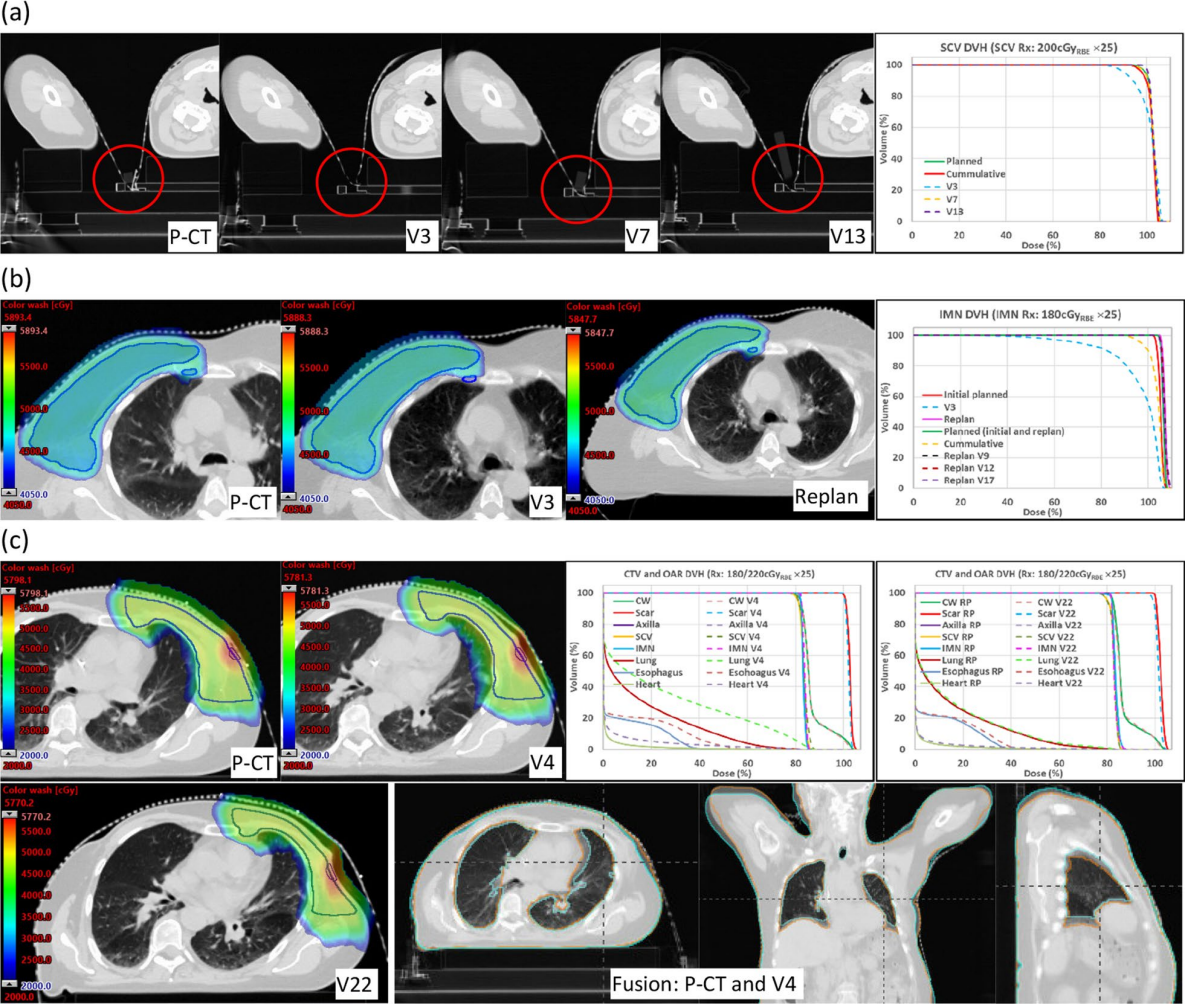
Masked patients with LDDs. **a** A patient with SCV LDD on V3 due to loose fixation point between upper arm and neck. SCV dose improved on V7 and V13 after the fixation point was secured. **b** The patient with IMN LDD on V3 due to inconsistent gap between mask and patient on P-CT and V-CT. Replan improved IMN dose for V9, V12, and V17. **c** A patient with increased gap between mask and skin on V-CTs compared to P-CT due to reduced CW tissue swelling, which caused lung and heart LDD. Replan improved lung and heart dose on V22. P-CT, planning computed tomography; V-CT, verification computed tomography; Vx, V-CT at fraction x; SCV, supraclavicular node; DVH, dose volume histogram; Rx: prescription; Gy_RBE_, Relative-Biological-Effectiveness dose in Gray; IMN, internal mammary nodes; CW, chest wall; CTV, clinical volume; OAR, organ at risk; RP, replan; LDD, large dose deterioration

The causes of LDDs for the 14 masked patients with at least one LDDs were mainly as follows: (1) The fixation point between upper arm and neck on the treatment side was not secured for P-CT and/or V-CT (10 patients); (2) There were noticeable gaps between mask and patient skin in treatment areas and the gaps were not consistent between V-CTs and corresponding P-CTs (5 patients); and (3) Breast/CW volume changed (2 patients). Figure 3a shows a patient with loose mask fixation point between upper arm and neck on V3 (Vx: V-CT at fraction x) caused SCV LDD. SCV dose improved after the fixation point was secured on V7 and V13. Figure 3b shows a patient had inconsistent gap between mask and skin in the treatment side on P-CT and V3, which caused IMN LDD. IMN dose improved with replanning for V9, V12 and V17. For this patient, cumulative dose IMN D95% improved by more than 16.0% compared to that on V3 but was still 8.1% lower than planned. Figure 3c shows a patient with decreased CW tissue swelling on V4 compared to P-CT, which caused ipsilateral-lung and heart LDD. Replanning resulted in improved ipsilateral-lung and heart dose on V22.

**Fig. 4:**
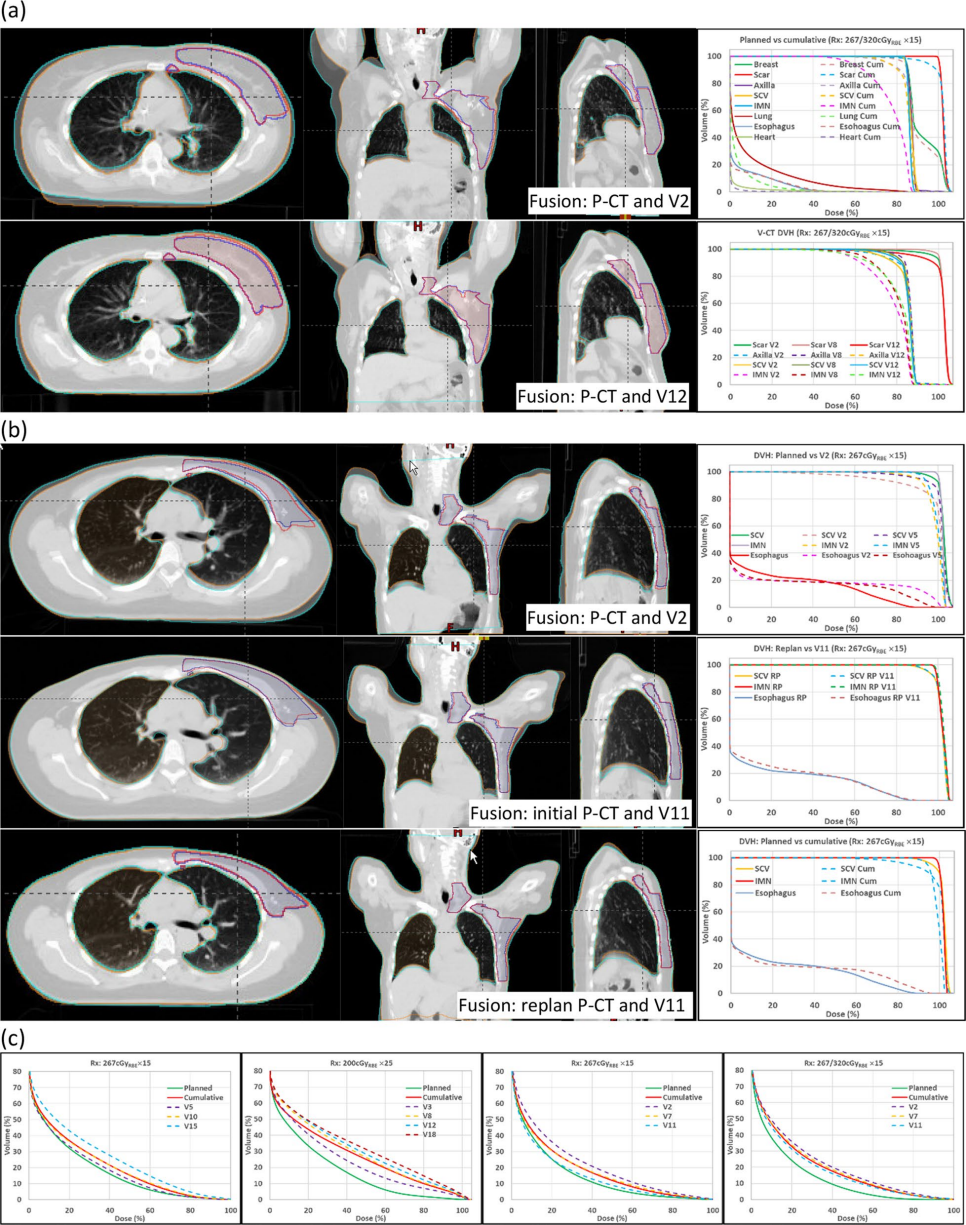
Maskless patients with cumulative dose CTV and OAR LDDs. **a** A patient with cumulative dose boost, SCV, and IMN D95% LDDs, **b** A patient with cumulative dose SCV and IMN D95% LDDs, and **c** Four maskless patients with cumulative dose lung LDDs. P-CT, planning computed tomography; V-CT, verification computed tomography; Vx, V-CT at fraction x; Rx: prescription; Gy_RBE_, Relative-Biological-Effectiveness dose in Gray; cum, cumulative; SCV, supraclavicular node; IMN, internal mammary nodes; RP, replan; CTV, clinical target volume; OAR, organ at risk

The causes of LDDs for the 18 maskless patients with at least one LDD were mainly as follows: (1) The arm and/or chin position variation, and/or breast/CW tissue deformation (18 patients); and (2) Breast/CW volume change (2 patients). Figure 4 shows selected DVHs and image fusions for the seven patients with CTV D95% and ipsilateral-lung V40% cumulative dose LDDs. The patient in Fig. 4a had all 3 V-CT and cumulative dose boost, SCV, and IMN CTV D95% LDDs. Fused image showed soft tissue deformation between P-CT and V-CTs. Setup improvement was attempted but physician did not order replan because the nodal irradiation was elective. Figure 4b shows a patient with SCV, IMN, and esophagus LDDs on V2 and V5. The patient was replanned starting with fraction 10. SCV, IMN, and esophagus dose DVH on V11 agreed well with replanned dose. Cumulative dose was improved compared to doses at V2 and V5 for SCV and IMN, however, D95% was still more than 5% worse than planned. Fused images revealed soft tissue deformation between initial P-CT and V-CTs, but more consistent soft tissue position between V11 and replan P-CT, which was also V5. Figure 4c shows the DVHs for the 4 patients who had cumulative ipsilateral-lung V40% LDDs. All V-CTs had worse ipsilateral-lung V40% compared to those on corresponding P-CTs.

## Discussions

To our best knowledge, this is the first study to comprehensively investigate the inter-fractional dose delivery reproducibility for proton therapy of breast cancer patients after breast conserving surgery or mastectomy with and without mask immobilization. On average, the CTV D95%, ipsilateral-lung V40%, esophagus D0.01cc, and heart mean dose evaluated on V-CTs at treatment position agreed with the corresponding planned dose parameters within a few percent for both masked and maskless patients.

Both immobilization methods have pros and cons. Mask immobilization has following advantages compared to maskless immobilization: 1. Mask immobilization can help to improve plan robustness by maintaining a reproducible breast shape and surface contour, reducing skin fold and smoothing sharp edges for soft tissues; 2. Well defined surface contour by mask allows easier identification of the soft tissue setup discrepancy when comparing V-CTs to P-CTs; and 3. Mask immobilization can help to limit large breathing magnitude in anterior surface in thoracic region and/or guide patient to do shallow or abdominal breathing. One of the concerns using mask to immobilize the breast is the soft tissue position inside mask may vary day to day. In this study each beam delivers a uniform dose to target, therefore, CTV gets planned dose disregard of the exact location of the soft tissue within mask if the shape defined by mask and marks around the breast on patient and mask are aligned within tolerance. Our results showed that if there were no gaps between mask and patient skin, the small variation of exact position of the breast tissue inside mask would not cause target and normal structure dose LDDs. The variation of the exact location of each part of the soft tissue inside the mask for each treatment fraction may average out hot/cold spots after multiple fractions of treatment. When the scar and nipple aligned to the marks on the mask marked at CT sim for each treatment session, clips alignment showed that boost volume alignment was also within tolerance for patients we treated with masks. Maskless immobilization has the following advantages compared to mask immobilization: 1. Maskless setup takes less device fabrication time and setup time at CT simulation and less setup time at each fraction of treatment; and 2. Maskless setup is consistent with photon radiation therapy setup so that both photon and proton treatment plans can be optimized on the same P-CT scan.

For masked patients, we applied 4-point mask with in-house added extra fixation point between patient upper arm and neck on the treatment side instead of thoracic mask because our initial study showed thoracic mask had difficulty to enclose large breast and had poor immobilization in shoulder and upper arm areas on the treatment side. However, with our method, therapists sometimes had hard time to secure the added fixation point between upper arm and neck at treatment. We have contacted the Orfit and a special designed mask with manufactured extra fixation point is possible in the future if there are reasonable demands.

For masked patients, mask would be taken off and reapplied if x-ray image showed bony structures and/or BBs were out of tolerance. For maskless patients, patient posture would be adjusted based on x-ray images to get setup within tolerance. If reapply mask or adjust patient posture won’t get setup within tolerance, a V-CT may be prescribed to evaluate dose distribution.

All V-CTs in this study were done in treatment room at treatment position after x-ray setup using CT-On-Rails except 16.7% of the 96 V-CTs for masked patients when CT-On-Rails were not available. The conclusion won’t change for this study if we exclude the V-CTs performed in CT sim rooms. The percentage of masked patients with LDDs are 20.3% and 22.0% with and without V-CTs in CT sim rooms included, respectively. When consistent mask application methods were used, we observed consistent bony structure and BB alignment on V-CT in CT sim room and x-ray images in treatment room. Therefore, V-CTs performed in CT sim rooms can represent the setup in treatment room if mask is applied consistently and we sometimes schedule V-CTs in CT sim room when CT-On-Rails is not available. For masked patients, when there are anatomy changes, there may be difficulties to get mask aligned or fastened or may see gaps between mask and patient skin at treatment setup that were not present at CT-sim. Under such circumstance, V-CT may also be prescribed for masked patients in CT sim room when CT-On-Rails in treatment room is not available. For maskless patients, we may need manipulate patient posture based on x-ray images during setup in treatment room. Therefore, we only perform V-CT for maskless patients using CT-On-Rails in the treatment room. For maskless patients, we don’t have soft tissue alignment problems as we have for masked patients when apply masks on patients. However, due to soft tissue variations are harder to be identified during treatment setup on x-ray images for maskless patients, we usually schedule weekly V-CTs for each patient in the treatment room with CT-On-Rails in advance.

To accurately evaluate inter-fractional dose variation and cumulative dose patient received, daily V-CT is needed. We don’t perform daily V-CT because acquiring daily V-CT is limited by imaging dose to patient and resource availability. This is a limitation of our study. The cumulative doses were calculated for patients with weekly V-CTs. The comparison of the V-CT to P-CT image registration and daily kV x-ray image to P-CT registration showed each V-CT was a reasonable representation of the patient position and anatomy for a few fractions of treatments close to the date the V-CT was performed. Patients with 1 or 2 V-CTs were not included for cumulative dose comparison because very small sample size may increase the margin of error. One of the maskless patients had 3 V-CTs for the first three weeks of the treatment but no V-CTs for last three weeks of treatment was not included in cumulative dose comparison either. This patient had increased ipsilateral-lung dose each week with the 3rd week ipsilateral-lung dose being highest. The patient was not included in cumulative dose comparison because using the 3rd weekly V-CT to represent the treatment of last 4 weeks could result in a cumulative dose that deviated too much from the actual dose the patient received.

Ideally, the dose uncertainty caused by setup uncertainty will be random and CTV/OAR doses fluctuate near the planned values for each patient during the treatment course so that the average dose over the course of treatment will be close to what was planned. Our data showed that the average dose parameter values calculated on V-CTs for all patients closely agreed to the planned values, consistent with random daily setup uncertainty. However, some patients had CTV/ORA LDDs on all V-CT and cumulative doses, suggesting that daily dose variation are not always randomized for each patient. For patients with CTV or OAR cumulative LDDs, the trend was all V-CT doses had dose deterioration for the same structures compared to the corresponding P-CT doses. Figure 4c showed four maskless patients with cumulative dose lung LDDs, whose V-CT lung V40% were all higher than the corresponding P-CT lung V40%. Though the average V-CT CTV/OAR dose parameters for all patients were close to those of the P-CTs, the improvement of the dose parameters for one patient won’t necessarily help another patient who had dose deterioration. In this study, there were more maskless patients showed lung and esophagus LDDs than masked patients.

A shortfall of our study was that the dose reproducibility only considered inter-fractional motion. Intra-fractional motion including respiratory motion effect was not evaluated. Due to the large target volume and frequency use of hypo-fractionated treatments, each proton breast treatment beam usually takes a few minutes to deliver at our clinic with the longest single beam delivery time was approximately 15 min. Due to the long beam delivery time, intra-fractional motion could also be a concern for proton therapy of breast cancer patients.

For our clinical practice, the LDD criterions used in this study are only what we use to investigate causes of dose changes to provide feedback for treatment setup improvements but not replan criterions. The replan decision was only based on treating physician’s clinical judgement. Among all replans in this study, two masked patients replanned without LDDs. The reason physician requested the replans for the two masked patient without LDDs were: 1. physician changed esophagus dose constraints in the middle of treatment; and 2. physician requested a replan for a bilateral patient whose V-CT right lung V40% increased by 4.2% (V-CT left lung V40% decreased by 0.8%), which was considered high for the patient.

We are cognizant that our data were from a single institution. We used the same planning strategies and delivery system for both group of patients so that the comparison was only between immobilization methods. Our data may not apply to other institutions depending on planning strategies and delivery methods.

## Conclusions

In this study, we compared inter-fractional dose reproducibility for proton treatment to breast/CW with and without mask immobilization after breast conserving surgery or mastectomy. On average, both setups achieved delivered CTV and OAR doses within a few percent of those of planned doses. However, patient immobilized with masks showed lower rate of CTV and OAR LDDs when V-CT and P-CT doses were compared. Dosimetric differences large enough to raise clinical concerns in either group were able to be addressed with replannings.

## Data Availability

The datasets used and/or analyzed during the current study are available from the corresponding author on reasonable request.

## Abbreviations

CT: Computed tomography
CTV: Clinical target volume
Cum: Cumulative
CW: Chest wall
D0.01cc: Dose to 0.01cc volume
D95%: Dose to 95% volume
DRR: Digitally reconstructed radiograph
DVH: Dose volume histogram
IMN: Internal mammary nodes
LDD: Large dose deteriorations
OAR: Organ at risk
P-CT: Planning simulation CT scan
PBS: Proton pencil beam scanning
RBE: Relative biological effectiveness
RP: Replan
Rx: Prescription
SCV: Supraclavicular node
SD: Standard deviation
V40%: Volume receiving 40% of the prescription dose
V-CT: Verification simulation CT scan
Vx: V-CT at fraction x
